# Aortic distensibility measured by pulse-wave velocity is not modified in patients with Chagas' disease

**DOI:** 10.1186/1477-5751-5-9

**Published:** 2006-06-12

**Authors:** Humberto Villacorta, Luiz Aparecido Bortolotto, Edmundo Arteaga, Charles Mady

**Affiliations:** 1 Heart Institute (InCor), University of São Paulo, Medical School, São Paulo, Brazil; 2 Hospital Pró-Cardíaco, Rio de Janeiro, Brazil

## Abstract

**Background:**

Experimental studies demonstrate that infection with *trypanosoma cruzi *causes vasculitis. The inflammatory lesion process could hypothetically lead to decreased distensibility of large and small arteries in advanced Chagas' disease. We tested this hypothesis.

**Methods and results:**

We evaluated carotid-femoral pulse-wave velocity (PWV) in 53 Chagas' disease patients compared with 31 healthy volunteers (control group). The 53 patients were classified into 3 groups: 1) 16 with indeterminate form of Chagas' disease; 2) 18 with Chagas' disease, electrocardiographic abnormalities, and normal systolic function; 3) 19 with Chagas' disease, systolic dysfunction, and mild-to-moderate congestive heart failure. No difference was noted between the 4 groups regarding carotid-femoral PWV (8.4 ± 1.1 vs 8.2 ± 1.5 vs 8.2 ± 1.4 vs 8.7 ± 1.6 m/s, P = 0.6) or pulse pressure (39.5 ± 7.6 vs 39.3 ± 8.1 vs 39.5 ± 7.4 vs 39.7 ± 6.9 mm Hg, P = 0.9). A positive, significant, similar correlation occurred between PWV and age in patients with Chagas' disease (r = 0.42, P = 0.002), in controls (r = 0.48, P = 0.006), and also between PWV and systolic blood pressure in both groups (patients with Chagas' disease, r = 0.38, P = 0.005; healthy subjects, r = 0.36, P = 0.043).

**Conclusion:**

Carotid femoral pulse-wave velocity is not modified in patients with Chagas' disease, suggesting that elastic properties of large arteries are not affected in this disorder.

## 

The elastic properties of the large arteries are important determinants of circulatory physiology [1]. Such properties can be assessed noninvasively by pulse-wave analysis, by ultrasound techniques, or by calculating the velocity of pulse-wave transit in a given arterial segment. The measurement of pulse-wave velocity (PWV) by noninvasive devices has been considered an index of arterial distensibility and stiffness. Indeed, using a modification of the Bramwell-Hill equation [2], it can be considered that distensibility is equal to the inverse of blood viscosity multiplied by the square of PWV. So, the carotid-femoral PWV is a recognized index of aortic distensibility and stiffness and has been shown to be an important predictor of cardiovascular events in many disorders, such as end-stage renal disease, arterial hypertension, diabetes, and ischemic heart disease [3-5].

Chagas' disease, or South American trypanosomiasis, is caused by the hemoflagellate *Trypanosoma cruzi *and is an important cause of heart disease in South and Central America. Patients with Chagas' disease have an early impairment of baroreflex function [6-8]. A possible mechanism responsible, at least in part, for this abnormality could be an impairment in arterial distensibility. Data obtained in experimental studies support this issue by demonstrating that acute infection by *trypanosoma cruzi *causes inflammatory lesions in large arteries, affecting both muscular and endothelial layers, besides an increased production of alpha tumoral necrosis factor and interleukin [9,10]. Although such alterations could hypothetically lead to a decreased distensibility of the large arteries in the chronic stage of Chagas' disease, this hypothesis has never previously been tested. Therefore, we sought to assess carotid-femoral PWV, a well-recognized index of aortic distensibility, in patients with different forms of Chagas' disease and to determine the clinical, echocardiographic, and functional parameters correlated with PWV in such patients.

## Methods

### Population

From July 1999 to August 2000, 53 patients with Chagas' disease (20 men and 33 women, mean age 49 ± 8.6 years) and 31 healthy volunteers (15 men and 16 women, mean age 45.3 ± 8.9 years) were studied. The diagnosis of Chagas' disease was based on positive serological reactions to Chagas' disease assessed by 2 methods, ELISA and immunofluorescence. On the basis of medical history, physical examination, roentgenogram of the thorax and electrocardiogram, all subjects were free of other cardiovascular or systemic disease. All subjects showed normal laboratory testing including a complete blood account, serum electrolytes, blood glucose, blood urea nitrogen, serum creatinine, total cholesterol, and triglyceride levels. A roentgenogram of the thorax was performed in all individuals. All patients with Chagas' disease underwent bidimensional echocardiography (Phillips System-ATL HDI 3000, 2–4 MHz transducer, Phillips, Bothell, WA) to assess systolic function. Patients with a left ventricle (LV) fractional shortening ≤25% were considered as having systolic dysfunction. In addition, we also evaluated the peak Vo_2 _(maximal oxygen consumption) and slope Ve/Vco_2 _values during a maximal treadmill test in 50 patients with Chagas' disease. Pulmonary ventilation and gas-exchange data were determined on a breath-by-breath basis with a computerized system (Model Vmax 229, Sensormedics, Yorbalinda, CA). The peak oxygen uptake was considered to occur at the end of the bicycle cardiopulmonary exercise test (ramp protocol with a 10- to 15-W increment every minute up to exhaustion), when the subject no longer maintained the bicycle velocity at 60 rpm.

The exclusion criteria were a prior history of systemic hypertension or current blood pressure ≥ 140 × 90 mm Hg, age above 65 years, history of diabetes mellitus or serum glucose above 126 mg/dL, total cholesterol above 240 mg/dL, serum creatinine above 1.4 mg/dL, chronic atrial fibrillation, concomitant severe chronic disease, and patients with functional class IV (NYHA) heart failure. The patients were classified into 3 groups: a) group 1 – 16 patients with an indeterminate form of Chagas' disease, characterized by positive serum Machado Guerreiro reaction and no clinical manifestations; b) group 2 – 18 patients with Chagas' disease, electrocardiographic abnormalities, and normal LV systolic function; c) group 3 – 19 patients with Chagas' disease, systolic dysfunction, and congestive heart failure (CHF). The clinical data and PWV measurements obtained from the 3 groups of patients were compared with those obtained in 31 healthy age-sex matched volunteers with no cardiovascular disease (group 4, control group). Among the patients with CHF (group 3), 4 were in NYHA functional class I, 12 were in class II, and 3 were in class III. Seven patients in this group were on digoxin, 7 were on furosemide, 11 were on hydrochlorothiazide, 15 were on an angiotensin-converting-enzyme (ACE) inhibitor, 5 were on spironolactone. No patient was on beta-blocker therapy. For ethical reasons, we decided not to withdraw the medications. However, to minimize the effects of ACE inhibitors and diuretics on arterial distensibility, patients were asked not to take the morning doses of such drugs on the day of diagnostic testing. Written informed consent was obtained from all subjects before the study, and the protocol was approved by the Medical Ethics Committee of the University of São Paulo, Medical School, São Paulo, Brazil.

### Carotid-femoral PWV measurements and blood pressure determination

The measurements were performed with the patient in the supine position. Brachial blood pressure was measured with a mercury sphygmomanometer after 15 minutes of rest. Phases I and V of the Korotkoff sounds were considered respectively as systolic and diastolic blood pressure. Two measurements 5 minutes apart were averaged. Pulse pressure was calculated as the difference between systolic and diastolic blood pressure.

After blood pressure determination, the PWV measurement was performed in a controlled environment at 22°C. We measured carotid-femoral PWV by using an automatic device, Complier (Colson, Garges les Gonesses, France), which allows on-line pulse-wave recording and automatic calculation of PWV with 2 transducers (TY 306, Fukuda, Tokyo, Japan), one positioned at the base of the neck for the common carotid artery and the other over the femoral artery. The PWV is automatically calculated as the distance between these points divided by the time the pulse wave takes to go from one point to another. At least 10 measurements were taken in each subject, and the average was used for the analysis. The validation of this automatic method and its reproducibility has been previously described [11].

The correlations between carotid-femoral PWV and the following parameters were also evaluated: age, systolic blood pressure, serum sodium, LV fractional shortening, LV end systolic and diastolic diameters, and peak VO_2_.

### Statistical analysis

Data are expressed as mean ± SD. The Student *t *test was used to compare normally distributed continuous variables. Chi-square (χ^2^) test was used to compare categorical baseline characteristics. Analysis of variance (ANOVA) was used for the comparison between the 3 groups of patients and the control group. Linear regression analysis (Pearson's analysis) was used to assess correlations of continuous variables.

The reproducibility of PWV measurements by the device used in our study has been described [11], and the accuracy is better if a consistent number of measurements are done. In our study, each patient had at least 10 measurements, and a mean value of these measurements was obtained only if the standard deviation was below 0.5 m/s. Based on other studies, the estimated number of subjects in each group to give a statistically significant power in the differences of PWV was at least 15 (all groups of our study had more than 15 subjects). A clinically significant difference between 2 PWV measurements is probably above 1 m/s. P < 0.05 was considered significant. All data were processed with SPSS System software.

## Results

### Baseline characteristics

Baseline demographic, clinical, laboratory, and echocardiographic parameters of the 4 groups are shown in the table 1. No difference was observed between the groups regarding demographic, clinical, and laboratory tests. Likewise, no difference was observed between the 4 groups regarding pulse pressure. Thus, the mean values of group 1 were 39.5 ± 7.6 mm Hg, group 2 were 39.3 ± 8.1 mm Hg, group 3 were 39.5 ± 7.4 mm Hg, and control group were 39.7 ± 6.9 mm Hg (P = 0.99). All echocardiographic measurements were significantly different and obviously impaired in group 3 that comprised patients with systolic dysfunction. Also, the peak VO_2 _was significantly decreased in group 3 patients with systolic dysfunction, in comparison with that in patients without heart failure.

**Table 1:** Baseline characteristics in control and Chagas' disease groups.

**Variables**	**Group 1 N = 16**	**Group 2 n = 18**	**Group 3 n = 19**	**Control n = 31**	**P value**
Age (years)	49.7 ± 5.9	47.4 ± 9.3	49 ± 10	45.3 ± 8.9	0.33
Male sex	6 (37.5 %)	6 (33.3 %)	8 (42 %)	15 (48.4 %)	0.30
Systolic blood pressure (mm Hg)	120.5 ± 6.8	118.9 ± 11.3	116.6 ± 11.3	118.1 ± 10.3	0.72
Diastolic blood pressure (mm Hg)	79.8 ± 2.9	80 ± 5	77.6 ± 5.8	76.7 ± 7	0.29
Heart rate (bpm)	74.6 ± 5.7	67.7 ± 7.6	74 ± 12	71.8 ± 10	0.13
Body mass index (Kg/m^2^)	25.8 ± 4.3	24.4 ± 3.3	23.4 ± 4.5	25.4 ± 3.7	0.26
Total cholesterol (mmol/L)	208 ± 20.8	197 ± 21	190 ± 34	196.3 ± 17.8	0.28
HDL (mmol/L)	46.4 ± 8.4	48.4 ± 9.8	46.8 ± 15.4	50.3 ± 9.8	0.75
LDL (mmol/L)	139 ± 20.3	130.4 ± 15.4	131.7 ± 27.6	130.8 ± 16	0.68
Triglycerides (mmol/L)	131.4 ± 59.6	117.1 ± 49.8	128 ± 4	128.5 ± 49.2	0.89
Hematocrit	41.2 ± 4	42 ± 3	40 ± 3	39.8 ± 2.9	0.20
Hemoglobin (g/dl)	14.4 ± 1.2	14.5 ± 0.7	14 ± 1.2	14 ± 0.8	0.37
Serum glucose (g/dL)	98 ± 9.5	93.3 ± 6.4	101 ± 12.7	93.4 ± 9.3	0.12
Serum sodium (mEq/L)	140 ± 1.6	139.7 ± 1.6	139.3 ± 2.6	139.7 ± 2	0.75
Blood urea nitrogen (mg/dL)	32.8 ± 5.3	33.8 ± 5	38.8 ± 13.6	32.7 ± 4.6	0.20
Serum creatinine (mg/dL)	0.95 ± 0.1	0.97 ± 0.2	1.05 ± 0.2	0.96 ± 0.1	0.25
End diastolic diameter (cm)	4.7 ± 0.43	4.87 ± 0.44	6.36 ± 1.09	_	< 0.00001
End systolic diameter (cm)	3.18 ± 0.39	3.18 ± 0.4	5.11 ± 1.2	_	< 0.00001
Ejection fraction	69.6 ± 5.2	70.3 ± 6.3	48.7 ± 11	_	< 0.00001
Fractional shortening	33 ± 3.9	33.7 ± 5	20.9 ± 6.7	_	< 0.00001
VO_2 _(ml/Kg/min)	20 ± 4.3	21.7 ± 7.3	14.9 ± 2.1	_	0.003

VO_2 _= peak oxygen consumption.

### PWV analysis

**Figure 1:**
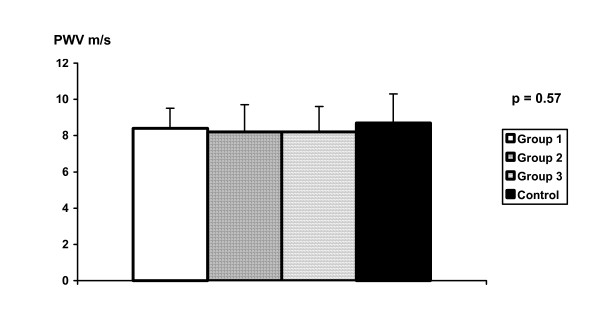
Pulse-wave velocity values in the 4 groups.

Carotid-femoral PWV values for the 4 groups of subjects are shown in figure 1. No difference was observed between the groups (P = 0.57). We also did ANOVA analysis by adjusting PWV values by age and mean blood pressure, and the differences between the 4 groups remained statistically nonsignificant. Mean values were respectively 8.4 ± 1.1 m/s in group 1 (range 6.7 to 11), 8.2 ± 1.5 m/s in group 2 (range 5.8 to 11.2), 8.2 ± 1.4 m/s in group 3 (range 5.9 to 11.9), and 8.7 ± 1.6 m/s in control group (range 5.7 to 14.3).

### Relationship between PWV and other variables

**Figure 2:**
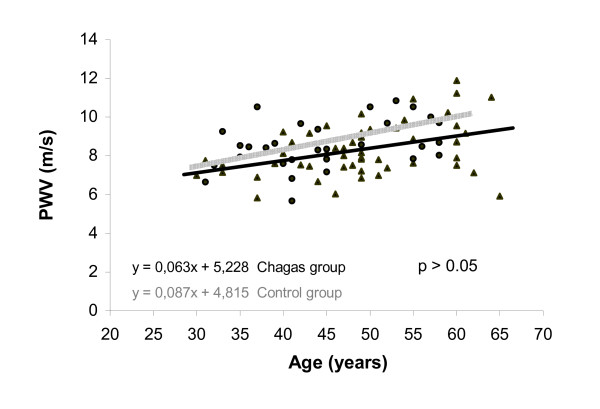
Relationship between pulse-wave velocity and age in Chagas' disease groups as compared with that in the control group.

**Figure 3:**
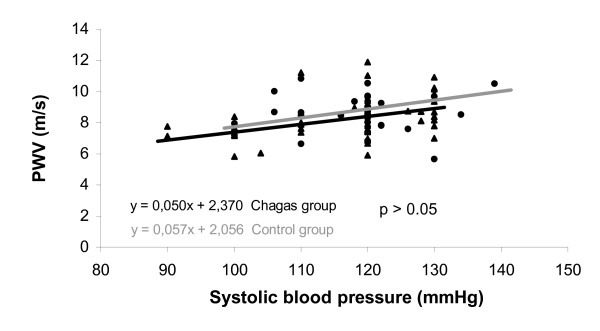
Relationship between pulse-wave velocity and systolic blood pressure in Chagas' disease group as compared with that in the control group.

**Figure 4:**
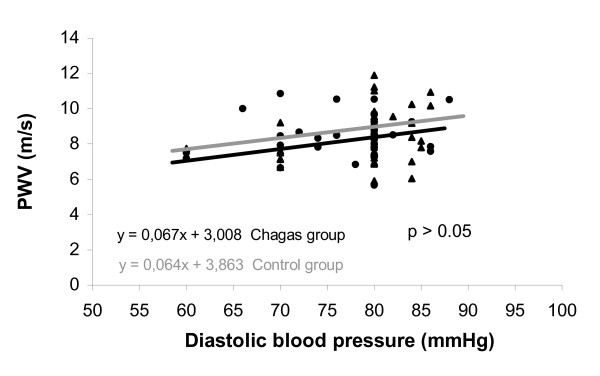
Relationship between pulse wave velocity and diastolic blood pressure in Chagas' disease group as compared with that in the control group.

Among the patients with Chagas' disease, no relationship was observed between carotid-femoral PWV and serum sodium (r = 0.172, P = 0.21), LV fractional shortening (r = -0.017, P = 0.90), LV end diastolic diameter (r = -0.16, P = 0.20), LV end systolic diameter (r = -0.12, P = 0.18), and peak VO_2 _(r = -0.059, P = 0.68). In both the control group (31 subjects) and the group of 53 patients with Chagas' disease, a positive, significant, and similar correlation was observed between carotid-femoral PWV and age (r = 0.42, P = 0.002, Chagas' disease group; and r = 0.48, P = 0.006, healthy subjects). Also we observed a significant correlation between carotid-femoral PWV and systolic blood pressure in both groups (patients with Chagas' disease, r = 0.38, P = 0.005; healthy subjects, r = 0.36, P = 0.043). When patients with Chagas' disease (n = 53) were compared with healthy subjects (n = 31), no differences were found regarding the slope, linear, and angular coefficients of the curve for both correlations between PWV and age and PWV and SBP (all P values above 0.05), as shown in figures 2 to 4.

## Discussion

This is the first study to noninvasively assess large artery distensibility by means of PWV analysis in Chagas' disease. We demonstrated that carotid-femoral PWV, a recognized index of aortic distensibility, is not modified in patients with Chagas' disease as compared with that in healthy subjects. Likewise, pulse pressure was not different among the 4 groups. These findings suggest that the elastic properties of large arteries are not affected by Chagas' disease, independently of the clinical manifestations of the disease.

Chagas' disease, or South American trypanosomiasis, is caused by the hemoflagellate *Trypanosoma cruzi *and has different forms of clinical manifestation, including asymptomatic or indeterminate form, electrocardiographic manifestations (right bundle-branch block, arrhythmias), gastrointestinal manifestations, and overt clinical heart failure. The pathological involvement of the heart in the chronic phase of Chagas disease is characterized by the presence of inflammatory infiltrates and focus of myocarditis associated with focal fibrosis, with a variable intensity. Experimentally, Chagas' disease causes vasculitis of the large arteries, affecting the muscular and endothelial layers [9,10]. This involvement could chronically produce modifications in the elastic properties of large arteries. However, in our study, aortic PWV was not different in patients with different forms of Chagas disease compared with that in healthy subjects. Although PWV is not a direct measure of arterial distensibility, several reports have considered it an important index of arterial stiffness and consequently, arterial distensibility. Some recognized cardiovascular risk factors could modify differently aortic distensibility and PWV measurement. However, the most important factors like age and blood pressure interfere similarly with both arterial distensibility and PWV measurement. In our study the correlations of PWV, age, and systolic blood pressure were statistically significant in both control and Chagas disease groups, supporting the data indicating that the influence of both blood pressure and age on vascular properties are not modified in patients with Chagas' disease. So, our data support the conclusion that aortic distensibility is not impaired in patients with different forms of Chagas' disease, even in those with heart failure.

Our results observed in patients without CHF coincide with those observed by Consolim-Colombo et al [12] who demonstrated that endothelial function was preserved in patients with Chagas' disease without CHF. Thus, it seems that functional properties of both small arteries, evaluated by endothelial function in the above-mentioned study, and large arteries, studied in our report are not affected by Chagas' disease.

Findings in patients with heart failure have been more contradictory, however. Arnold et al [13] showed that patients with mild to severe CHF had a significantly higher brachial PWV than healthy subjects had, even considering that patients with moderate to severe CHF were on vasodilator drugs. In another study, Giannattasio et al [14] also observed a decrease in radial artery compliance assessed by high-resolution ultrasound in 25 patients with CHF. On the other hand, Eliakim et al [15] and Merillon et al [16] did not observe any alterations in aortic PWV invasively assessed in patients with CHF. It is possible that such disparities may be due to differences in the severity of CHF present in the patients studied and also to the different methods used to assess arterial distensibility. More recently, Mitchell et al [17] has demonstrated that the central pulsatile load was increased in CHF, but, in contrast, distal (muscular) conduit vessels tended to be less stiff (lower carotid radial PWV) in the same patients with CHF. The increased functional stiffness of the central conduits in CHF observed in Mitchell's study is not apparent in global measures such as augmentation index or total artery compliance, probably explained by the contrasting changes in central and peripheral conduits. In our study, the absence of modifications in carotid pulse-wave velocity in Chagas' disease even in the presence of mild to moderate CHF, could be partially explained by the results of Mitchell et al [17].

Three possible mechanisms may be responsible for modifications in arterial distensibility in patients with CHF: increased plasma or tissue concentrations of a number of vasoconstrictor substances secondary to enhanced sympathetic drive; a reduction in the shear endothelium stress and secretion of endothelial relaxing factors as a consequence of a reduction in cardiac contractility and output; and arterial wall edema and stiffness due to sodium and water retention [18]. All these mechanisms are more intense in patients with severe CHF, patients who were excluded in our study so as not to interfere in a possible effect of Chagas' disease on the PWV. Thus, as we excluded patients with severe CHF, and also patients with ischemic heart disease or hypertension, situations that knowingly modify PWV, our results suggest that the presence of mild to moderate heart failure per se in patients with Chagas' disease does not alter the elastic properties of great arteries. However, as we do not assess other vascular functional properties like endothelial function in these patients, it is not possible to totally exclude vascular modifications in patients with Chagas disease and heart failure.

The lack of differences in carotid-femoral PWV between healthy subjects and the different groups with Chagas disease could be due to type II (beta) statistical error frequently observed in a study such as this involving a small number of patients. However, we can observe that the standard deviation of PWV measurements is very small, and even considering a greater statistical significance (0.10) the values remained not different among the groups.

In both the control healthy group and patients with Chagas disease, a positive and similar relationship between PWV and age and between PWV and systolic blood pressure was observed, as described previously [10]. We did this analysis to verify whether the aging and blood pressure influence on aortic distensibility could be modified by the presence of Chagas disease. Thus, Chagas' disease seems not to accelerate the arterial stiffening secondary to aging or elevated blood pressure.

One limitation of our study must be addressed. For ethical reasons, we did not withdraw diuretic and vasodilator drugs used by patients with CHF. Therefore, it would be possible that such drugs may have had a favorable effect on PWV, leading to a "pseudonormalization" of a modified carotid-femoral PWV in such patients.

In summary, the present study indicates that carotid-femoral PWV is normal in patients with Chagas' disease, despite the presence of electrocardiographic abnormalities or mild heart failure, suggesting that large artery distensibility is not primarily affected in this disease.
